# Sagittal Relationship between the Maxillary Central Incisors and the Forehead in Digital Twins of Korean Adult Females

**DOI:** 10.3390/jpm11030203

**Published:** 2021-03-13

**Authors:** Seoung-Won Cho, Soo-Hwan Byun, Sangmin Yi, Won-Seok Jang, Jong-Cheol Kim, In-Young Park, Byoung-Eun Yang

**Affiliations:** 1 Division of Oral and Maxillofacial Surgery, Hallym University Sacred Heart Hospital, Anyang 14068, Korea; kotneicho@gmail.com (S.-W.C.); purheit@daum.net (S.-H.B.); queen21c@gmail.com (S.Y.); wionnk@naver.com (W.-S.J.); ddskjc@hanmail.net (J.-C.K.); 2 Graduate School of Clinical Dentistry, Hallym University, Chuncheon 24252, Korea; park.iy2875@gmail.com; 3 Institute of Clinical Dentistry, Hallym University, Chuncheon 24252, Korea; 4 Daegu Mir Dental Hospital, Daegu 41940, Korea; 5 Division of Orthodontics, Hallym University Sacred Heart Hospital, Anyang 14068, Korea

**Keywords:** single upper central incisor, face, computer-assisted three-dimensional imaging, cone-beam computed tomography

## Abstract

Objective: Digital twins of adult Korean females were created as a tool to evaluate and compare the sagittal relationship between the maxillary central incisors and the forehead before and after orthodontic treatment. Methods: Digital twins were reconstructed for a total of 50 adult female patients using facial scans and cone-beam computed tomography (CBCT) images. The anteroposterior position of the maxillary central incisor and the forehead inclination were measured. Results: The control group presented a mean of 6.7 mm for the sagittal position and 17.5° for forehead inclination. The study group showed a mean of 9.3 mm for the sagittal position and 13.6° for forehead inclination. Most Korean females seeking orthodontic treatment had their maxillary central incisor anterior to the glabella. In contrast, fewer Korean females who completed their orthodontic treatments had their maxillary central incisor anterior to the glabella. Furthermore, patients who had completed the orthodontic treatment were more likely to have the maxillary central incisor between the forehead facial axis and glabella. Conclusion: The use of digital twins for three-dimensional (3D) analysis of the profile implies a high clinical significance. In addition, as the facial profile of Koreans is different from that of Caucasians, careful consideration should be made when setting treatment goals for the anteroposterior position of the maxillary central incisors.

## 1. Introduction

An accurate diagnosis of a facial profile must be made prior to any orthodontic treatment. Earlier investigators, including Downs, Steiner, Tweed, Sassouni, and Ricketts focused on the use of cephalometric analysis to evaluate facial profile in a more accurate and reproducible manner [1,2,3,4,5,6]. Owing to its high applicability in dental clinics, cephalometric analysis has been established as a tool routinely used for profile diagnosis. In this technique, skeletal or soft tissue landmarks are used to define points, lines, and/or planes and quantify the positions of facial structures. However, the use of such landmarks can be unreliable, because the identification of the landmarks can vary between observers, and errors can arise from the head postures of the individuals [7,8,9]. The two-dimensional (2D) radiographic images may also be affected by magnification, craniofacial asymmetry, and the superimposition of anatomical structure, enhancing the imprecision for the assessment [10]. In addition, good facial harmony exists within a broad spectrum of cephalometric values [11]. Furthermore, with regard to facial esthetics, cephalometric analysis in isolation cannot consider every anomaly that is clinically presented [12]. Therefore, complete reliance on cephalometric analysis may lead to unexpected esthetic problems.

With contributions to the technological advancement in three-dimensional (3D) imaging, cone-beam computed tomography (CBCT) allows for routine 3D assessment of a facial profile. It is true that routine CBCT recording still has several limitations, such as higher radiation exposure compared to lateral cephalogram and artifacts driven from the metallic material or motion [13,14]. However, advantages of the 3D-generated cephalometry over conventional 2D cephalometry include the ability to perform various measurements in different planes [15]. In addition, it does not require strict standardization of the head position. Two-dimensional imaging can display an inaccurate representation of anatomy caused by head posture, rotational, and geometric errors [16]. Another limitation of the 2D imaging technique is apparent in complex cases such as orofacial clefts and syndromes presenting craniofacial deformity [17]. The conventional 2D analysis can be unpredictable, because cephalometric measurements are easily distorted in the presence of facial asymmetry [15]. Most importantly, the 3D method enables identification and reorientation of the bilateral landmarks, which is not available in 2D cephalometry [18].

Moreover, the introduction of the facial scanning technique has enabled the 3D reconstruction of the patient by assembling and rebuilding every data obtained. The data, reconstructed in the virtual environment to reproduce reality, which provides answers to a range of clinical questions, are referred to as digital twin [19]. Digital twins can be used to predict the outcome of specific procedures. It can help to determine the optimal treatment option for specific patients. Although 3D imaging techniques add precision to an evaluation of craniofacial morphology, however, most assessment protocols are built on the concepts of 2D cephalometry [20,21]. 

As a method to achieve an attractive facial profile, Andrews proposed the philosophy of the Six Elements of Orofacial Harmony [22]. He defined the second element as the anteroposterior position of the jaw and used it as a landmark for evaluating the sagittal position of the maxillary central incisors in the profile. He claimed that the six keys to orofacial harmony should work equally well for patients regardless of sex, age, or race. While many researchers have studied the relationship between the forehead and maxillary central incisors in Caucasian males, females, and African-American females, little has been studied in Korean females [23,24,25,26]. Even in existing research, the analysis was made simply on the profile photograph, which was aligned in a rather variable position. 

The purpose of this study was to create a digital twin of the patient using CBCT and facial scan to evaluate and compare the sagittal relationship of the maxillary central incisors to the forehead in adult Korean females before (study group) and after orthodontic treatment (control group). To the best of our knowledge, this is the first study to investigate the anteroposterior relationship between the maxillary central incisors and the forehead in Korean females in 3D using a digital twin created with facial scan and CBCT. 

## 2. Materials and Methods

### 2.1. Sample

Facial scan files and CBCT images were obtained from 50 adult female patients who visited Hallym Sacred Heart Hospital between September 2015 and September 2020. The facial scan images were acquired using RAYface^®^ (Raymedical, Seongnam, Korea), while CBCT was taken with Asahi Alphard 3030^®^ (Asahi Roentgen Ind., Co., Ltd., Kyoto, Japan). The CBCT scan was acquired in C-mode with an imaging volume of 200 × 178 mm and a voxel size of 0.39 mm. The scanning parameters were fixed at 80 kVp, 5 mA, and 17 s for all patients. The patient inclusion criteria were as follows: (1) adult females with completely developed jaws and (2) Korean ethnicity. The exclusion criteria were as follows: (1) cleft lip and palate or syndromic diagnosis, (2) incomplete records, (3) history of another orthodontic treatment, and (4) hypodontia. The control group was composed of 25 facial scan files and CBCT images of the patients who had completely finished the orthodontic treatment.

Of the 96 samples who had completed orthodontic treatment between September 2015 and September 2020, photos of 25 patients were selected as a control sample when three dentists unanimously agreed that the patient showed an esthetically pleasing profile. It was considered as an attractive profile when the lips and chin were in harmony with the rest of the face [27,28]. The three dentists were not informed of the preexisting skeletal or dental relationships or the specific treatment procedure the patient had undergone. The mean age of the group was 23.9 years. The study group was composed of the remaining 25 facial scan files and CBCT images of the patients who first visited the orthodontic treatment clinic. The mean age of the group was 23.0 years. None of the patients underwent orthognathic surgery for orthodontic treatment. The study protocol was approved by the Hallym University Sacred Heart Hospital Institutional Review Board (IRB No. 2019-08-003-001). The IRB approved this retrospective study, and all patient data were anonymized and de-identified before the analysis.

### 2.2. Profile Analysis

**Figure 1:**
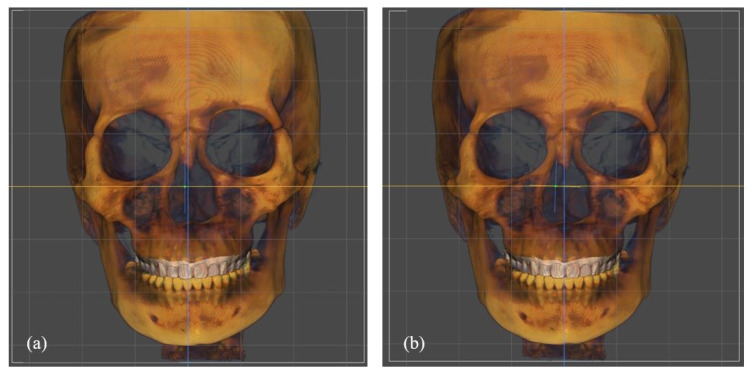
Reorientation process of the skull. (**a**) Before the reorientation, the coronal view reveals a canting of the skull. (**b**) After the reorientation.

**Figure 2:**
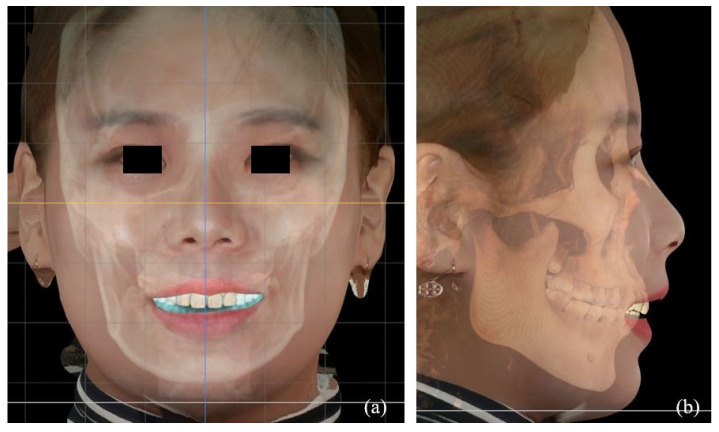
A digital twin reconstructed by the fusion of facial scan and cone-beam computed tomography images. (**a**) Coronal view of the face. (**b**) Sagittal view of the face.

The CBCT images in digital imaging and communications in medicine (DICOM) format and facial scan files in standard tessellation language (STL) format were imported to the FaceGide^®^ (Megagen Co., Ltd., Daegu, Korea) program. The reorientation of the skull started with setting a horizontal plane to involve the orbitale on both sides and the right porion (Figure 1). The orbital rims on both sides were matched on the sagittal plane. Then, the sagittal plane was set based on the Frankfort horizontal plane by identifying the porion and orbitale on CBCT. A midsagittal plane was then set based on the soft tissue. Superimposition of the CBCT and 3D facial scan was carried out on the identical program to create a digital twin (Figure 2). The facial replica was aligned with reference to the midsagittal plane to exhibit the right profile of the face.

To evaluate the profile, soft tissue reference points were marked with reference to the facial scan image. For a “straight” forehead shape, the trichion was marked, while it was replaced with the superion for “rounded or angular” foreheads. The glabella was identified and marked. Subsequently, a line was drawn by connecting the glabella to the trichion or superion. The midpoint of this line was marked as the forehead facial axis (FFA) point. The maxillary central incisor was marked as the facial axis (FA) point. Brief definitions of the defined landmarks are as follows: the trichion is defined commonly as the hairline and the most superior aspect of the forehead on a flat forehead; the superion is the most superior aspect of the forehead when the forehead shape is either rounded or angular; the glabella is defined as the most prominent midpoint between the eyebrows, and the FA point of the maxillary central incisor is the landmark point that separates the gingival half of the clinical crown from the occlusal half.

**Figure 3:**
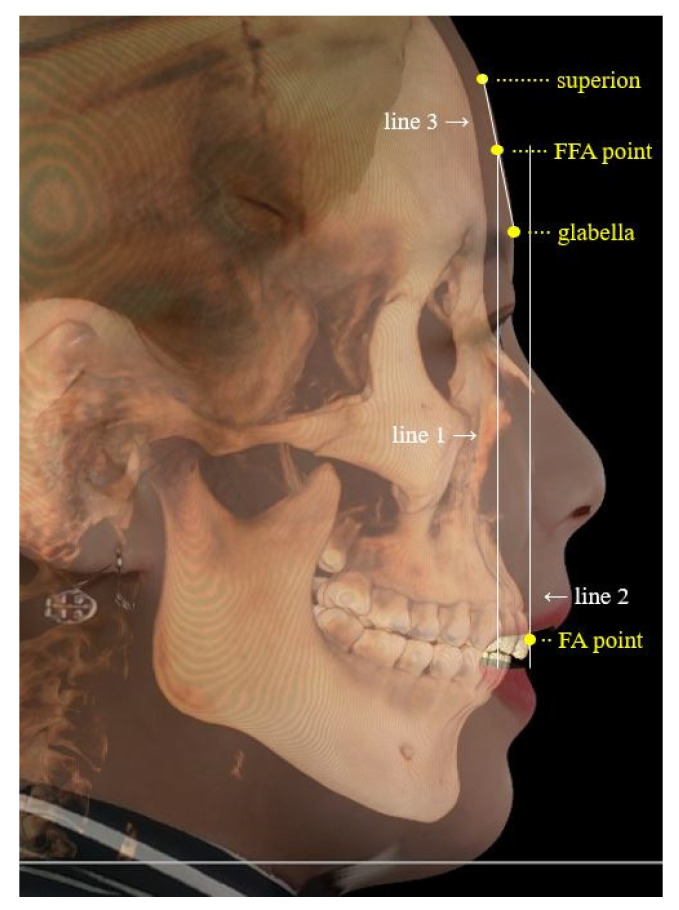
Profile analysis on the program. Each reference point is identified in yellow. Line 1 is the vertical line constructed from the FFA point. Line 2 is the vertical line constructed from the FA point. Line 3 is formed by connecting the superion and the glabella. FA: facial axis; FFA: forehead facial axis.

Consequently, two vertical lines identified as lines 1 and 2 were constructed from the FFA point and FA point, respectively. Line 3, constructed by connecting the glabella to the superion or the trichion, was added to measure forehead inclination (Figure 3). The angular measurement was performed using a protractor tool in the program. The anteroposterior relationship of the maxillary central incisors to the forehead was measured as the distance between lines 1 and 2 using the software’s ruler tool (Figure 3). A positive value was assigned when the maxillary central incisors (line 2) were positioned anterior to the FFA point (line 1), and negative when posterior.

### 2.3. Statistical Analysis

Descriptive and comparative statistical analyses were performed using the Statistical Package for Social Sciences (SPSS version 23.0, IBM Co., Armonk, NY, USA). The means, standard deviations (SD), and ranges were calculated to describe the maxillary central incisor position relative to the forehead and the forehead inclination in both groups. The means for both groups were compared using an independent *t*-test. Herein, *p* values of ≤0.05 implied significant differences. Two-tailed Pearson correlation analysis was performed between the maxillary central incisor position and forehead inclination for both samples to identify the correlation.

### 2.4. Error Analysis

The entire process from the reorientation of the CBCT through measurement was repeated by the same examiner. The systematic error between the first and second measurements was calculated using the interclass correlation coefficient of reliability (R).

## 3. Results

Intra-examiner reproducibility was verified using the interclass correlation coefficient of R. An R-value greater than 0.90 was considered to indicate high reliability. Table 1 shows the mean and SD between the first and second measurements and the reliability of the measurements in the first and second measurements in the incisor position and forehead inclination. The reliability was significantly high for every measurement.

The sagittal position of the maxillary central incisors relative to the forehead, which was manifested as the distance between lines 1 and 2, is shown in Table 2. For the control group, the mean sagittal position of the maxillary central incisors relative to the FFA point of the forehead was 6.7 mm, with a standard deviation of 4.1 mm. The distance value ranged from 0 to 14.8 mm. The maxillary central incisor position in the study group had a mean value of 9.3 mm with a standard deviation of 4.9 mm, ranging from 1.2 to 20.4 mm. The sagittal position of the maxillary central incisors relative to the FFA point of the forehead was significantly different between the two groups (*p* < 0.05; Table 3).

**Figure 4:**
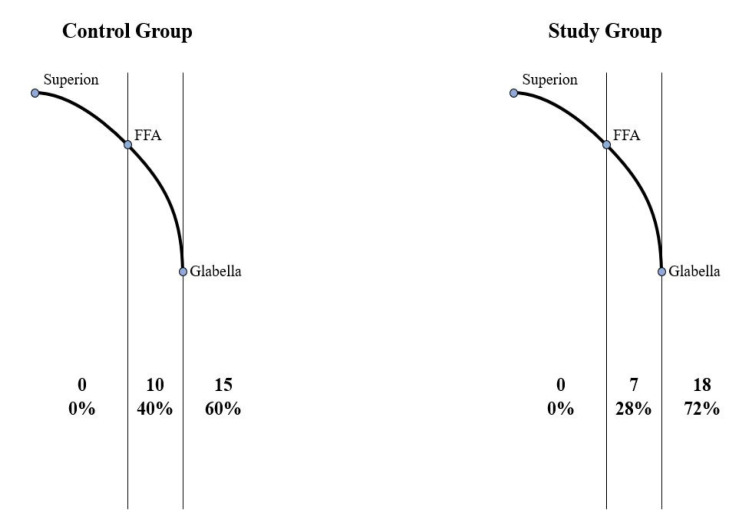
Distribution of the anteroposterior maxillary central incisor positions relative to the forehead for the control and study groups. FFA: forehead facial axis.

The distribution of the maxillary central incisor position is shown in Figure 4. In the control group, most of the patients had central incisors anterior to the glabella (60%), while the others had them at or between the FFA and the glabella (40%). None of the patients had their central incisors posterior to the FFA point. On the other hand, more patients in the study group showed maxillary central incisors anterior to the glabella (72%). The other patients had central incisors at or between the FFA and the glabella. 

Forehead inclination, calculated as the angle between lines 3 and 1, is presented in Table 4. The forehead inclination in the control group ranged from 3.7 to 25.5° with a mean angle of 17.5° and a standard deviation of 5.6°. In the study group, the forehead inclination ranged from 1.2 to 19.4° with a mean of 13.6° and a standard deviation of 5.9°. The difference between the groups was significant (*p* < 0.05, Table 4).

Table 5 shows the correlations between the maxillary central incisor position and the forehead inclination in the control and study groups. When it comes to the control group, the maxillary central incisor position and forehead inclination showed correlations that were not significant (r = 0.384, *p* = 0.058). For the study group, the maxillary central incisor position and the forehead inclination were also not significantly correlated (r = 0.379, *p* = 0.062).

## 4. Discussion

Considering that the maxillary central incisors are an integral part of the face, an orthodontist should evaluate the facial profile with the maxillary incisors included. Similar to the evaluation of the dental and facial midlines, another facial landmark is required to assess their positions in the sagittal plane when the maxillary central incisors are displayed. This study aimed to evaluate the anteroposterior position of the maxillary central incisors in relation to the forehead in adult Korean females.

Despite various investigations over several decades, no analysis has been found to be completely predictable in identifying the ideal anteroposterior position of the maxilla. Steiner suggested the use of the angle designated as Sella-Nasion-A point (SNA) to determine the sagittal position of the maxilla in comparison to population norms [29]. However, SNA measurements can vary according to the length and position of the skull base, especially in patients with dentofacial deformities. For this reason, linear analyses have been developed to overcome the limitations of angular measurements. McNamara, for example, proposed the use of the distance from the Nasion perpendicular line to the A-point in the natural head position [30]. However, cephalometric analysis alone was unreliable in patients with dentofacial deformities and did not necessarily correspond to facial esthetics [31,32]. Using the forehead as a primary landmark for assessing the positions of maxillary central incisors can help avoid potential fallacies of relying merely on cephalometric analysis. 

The use of CBCT is common as a part of routine orthodontic records in clinics, owing to its advantages, including lower cost and the amount of radiation exposure as well as good image quality compared to multidetector computed tomography (MDCT) [33,34]. Various dental imaging software programs have also been developed to produce 2D cephalograms based on CBCT images [35]. Meanwhile, many researchers have conducted studies to compare measurements of virtual cephalograms generated from CBCT images with those of 2D cephalograms. Some studies have verified the accuracy and reproducibility of the virtual lateral cephalometric radiographs derived from CBCT [36,37]. Therefore, 3D CBCT has been used for the profile analysis in this study because of its high practicality.

Previous methods for the profile assessment have been limited to 2D, commonly using cephalogram, photograph, or the combination of both [38,39]. In this conventional method, the reorientation process usually requires more than one photograph. However, in this study, the 3D analysis was performed based on the fused images of CBCT and 3D facial scans. Therefore, reducing the height of the beam when a full head view was not required and reducing the imaging parameters as low as possible was allowed. In other words, integration of the facial scan image to the CBCT image facilitated the identification of the glabella, trichion, or superion, which unnecessitated the wide field of view (FOV), including the entire skull [40,41]. In addition, this allowed for the identification of the true midsagittal plane, which facilitated the accurate reorientation process of the digital twin. In particular, RAYface^®^ (Raymedical, Seongnam, South Korea) used in this was based on quick flash 3D scan technology, which enabled the acquisition of the 3D face data without distortion of patients whose movements cannot be restrained for an extended time, especially those with uncomfortable jaws [42,43]. The advantages of using digital twins have been verified in orthognathic surgery as well [44]. In the computer-aided surgical simulation system, digital twins have been used for diagnosis and virtual surgery, resulting in a successful outcome in the actual surgery. 

Various methods have been proposed to enhance the reproducibility of the CBCT reorientation process. In this study, the orbits were used as the primary reference structures, because they are claimed to be relatively symmetrical and capable of providing standard reference landmarks and planes in an intuitive manner [45]. The adjustment of both orbital frames solves the canting as well as yawing simultaneously. Then, the sagittal plane can be set based on the Frankfort horizontal plane or the natural head position. The former was opted in this study, since its landmarks were easily detected on CBCT. Then, the midsagittal plane was set on the coronal view of the soft tissue to make an assessment based on soft tissue landmarks. 

The anteroposterior position of the maxillary central incisors relative to the forehead has been investigated in adult Caucasian females. In his study, Andrews reported that most (64%) of the patients had the maxillary central incisors posterior to the FFA point before orthodontic treatment. However, contradictory results have been found in this study. Most of the Korean female patients seeking orthodontic treatment had the maxillary central incisors anterior to the glabella. This may be attributed to the different cephalometric measurements in various ethnic groups [46]. An earlier study revealed that the maxillary incisors of Koreans are more protrusive and labially inclined than those of Caucasians [47]. It has also been reported that Koreans have more protruded lips. In addition, the prevalence of malocclusions varies in different communities. Salonen et al. found that the distribution of malocclusions in Swedish Caucasians was 71, 23, and 5% for Angle’s Classes I, II, and III, respectively [48]. Burgersdijk reported that malocclusions in Dutch adults consisted of 69% for Class I, 28% for Class II, and 2% for Class II [49]. Similar results have been found in Australian Caucasian adults, with 67.1, 28.7, and 4.2% for Class I, II, and III, respectively [50]. On the other hand, the prevalence of malocclusions in Korean ethnicity was 60.5% for Class I, 21% for Class II, and 9.4% for Class III [51]. Another investigation in Koreans showed a similar distribution of 63.3, 8.7, and 10.8% for Classes I, II, and III, respectively [52]. 

There may be some potential limitations to this study. The primary limitation of this study was the small sample size. This may have affected the statistical outcome; therefore, considerations should be made in the generalization of results. Further studies with larger sample sizes are expected to be conducted in the future. 

## 5. Conclusions

The forehead has been proven to be a useful landmark for assessing the facial profile in Caucasians. As the facial profile of Koreans is different from that of Caucasians, careful consideration should be made when setting treatment goals for the anteroposterior position of the maxillary central incisors. Furthermore, setting the locations for the maxillary central incisors with the use of digital twins that are reoriented based on CBCT and integrated with 3D facial scan images implies a distinguishing clinical significance.

Most (72%) of the adult Korean females seeking orthodontic treatment examined in this study had maxillary central incisors positioned anterior to the glabella. The correlation between the positions of the maxillary central incisors and the forehead inclination was not significant.

Comparatively fewer (60%) adult Korean females who had completed orthodontic treatments had maxillary central incisors positioned anterior to the glabella. More samples (40%) in the control group had maxillary central incisors positioned between the FFA and the glabella than in the study groups (28%). The correlation between the positions of the maxillary central incisors and forehead inclination was not significant.

## Figures and Tables

**Table 1:** Measurement error analysis.

Group	Measurement	Mean	SD	R ^1^	*p*-Value
Control group	Incisor position	7.28	0.75	0.96	<0.001 *
(*n* = 25)	Forehead inclination	17.62	0.06	0.91	<0.001 *
Study group	Incisor position	9.42	0.04	0.99	<0.001 *
(*n* = 25)	Forehead inclination	14.16 ^1^	0.65	0.92	<0.001 *

^1^ R refers to reliability. * significance set at <0.05. SD: standard deviation.

**Table 2:** Anteroposterior position (mm) of the maxillary central incisors relative to the forehead’s forehead facial axis point (distance between lines 1 and 2).

Group	Mean	SD	Minimum	Maximum
Control group (*n* = 25)	6.7	4.1	0	14.8
Study group (*n* = 25)	9.3	4.9	1.2	20.4

SD: standard deviation.

**Table 3:** Anteroposterior position of the maxillary central incisors and the forehead inclination in the control and the study groups.

Measurement	Control	Study	*p*-Value
Position, mm	6.7	9.3	0.04
Forehead inclination, °	17.5	13.6	0.02

**Table 4:** Forehead inclination (angle between lines 3 and 1, °).

Group	Mean	SD	Minimum	Maximum
Control group (*n* = 25)	17.5	5.6	3.7	25.5
Study group (*n* = 25)	13.6	5.9	1.2	19.4

SD: standard deviation.

**Table 5:** Correlations between incisor position and forehead inclination.

Group	Position, mm	Inclination, °	r ^1^	*p*-Value
Control group (*n* = 25)	6.7	17.5	0.384	0.05
Study group (*n* = 25)	9.3	9.42 ^1^	0.379	0.06

^1^ r refers to correlation coefficient.
